# Parvalbumin and parvalbumin chandelier interneurons in autism and other psychiatric disorders

**DOI:** 10.3389/fpsyt.2022.913550

**Published:** 2022-10-12

**Authors:** Pablo Juarez, Verónica Martínez Cerdeño

**Affiliations:** ^1^Institute for Pediatric Regenerative Medicine (IPRM), Shriners Hospital for Children and UC Davis School of Medicine, Sacramento, CA, United States; ^2^Department of Pathology and Laboratory Medicine, UC Davis School of Medicine, Sacramento, CA, United States; ^3^MIND Institute, UC Davis School of Medicine, Sacramento, CA, United States

**Keywords:** chandelier cell, parvalbumin, autism, schizophrenia, interneuron

## Abstract

Parvalbumin (PV) is a calcium binding protein expressed by inhibitory fast-spiking interneurons in the cerebral cortex. By generating a fast stream of action potentials, PV+ interneurons provide a quick and stable inhibitory input to pyramidal neurons and contribute to the generation of gamma oscillations in the cortex. Their fast-firing rates, while advantageous for regulating cortical signaling, also leave them vulnerable to metabolic stress. Chandelier (Ch) cells are a type of PV+ interneuron that modulate the output of pyramidal neurons and synchronize spikes within neuron populations by directly innervating the pyramidal axon initial segment. Changes in the morphology and/or function of PV+ interneurons, mostly of Ch cells, are linked to neurological disorders. In ASD, the number of PV+ Ch cells is decreased across several cortical areas. Changes in the morphology and/or function of PV+ interneurons have also been linked to schizophrenia, epilepsy, and bipolar disorder. Herein, we review the role of PV and PV+ Ch cell alterations in ASD and other psychiatric disorders.

## Parvalbumin functional properties

Parvalbumin is a high affinity Ca^2+^ binding protein that acts as a buffer to sequester calcium in the cytoplasm of inhibitory neurons (1). Parvalbumin binds Ca^2+^ with high affinity (K_D, Mg_ ~ 5–100 uM) and to Mg^2+^ with medium affinity (K_D, Mg_ ~ 5–500 uM). PV is considered a “slow buffer” molecule due to its binding kinetics to those two ions, along with its slow association and dissociation rate (2). Action potentials in fast-spiking PV+ neurons are characterized by high frequency firing rate with minimal adaptation, narrow AP half width, short membrane time constant, and large afterhyperpolarization (3–5). PV+ interneurons exhibit high depolarized resting membrane potentials and small action potential amplitudes (6). Expression of unique voltage-gated Na^+^ and K^+^ channels contribute to the high-frequency repetitive firing patterns in PV+ interneurons (7, 8). By generating such a fast stream of action potentials, PV+ interneurons evoke a quick and stable GABA inhibitory postsynaptic current to pyramidal neurons that is characterized by fast rise and decay phases. This fast synchronization of network activity is responsible for the generation of high frequency gamma oscillations (9–11) that are associated with high level cognitive functions such as attention, memory, and perception (12–17). *In-vivo* optogenetic manipulation of PV+ interneuron activity is sufficient to produce changes in the network oscillations of the cerebral cortex of rats. Synchronous activity of PV+ cells amplify gamma oscillation and *in-vivo* inhibition of PV+ cells suppress gamma oscillations (10, 18). During firing, PV buffers intracellular Ca^2+^ levels back to normal physiological levels (19). The high abundance of mitochondria is the product of the substantial energy input required for PV+ neurons to sustain their characteristic high frequency firing patterns (20, 21). This increases their vulnerability to developing metabolic stress through the release of free radicals (22). Specifically, loss of PV leads to the pathological accumulation of mitochondrial Ca^2+^ levels that results in disruption of the electron transport chain (23). This leads to a buildup of intracellular reactive oxygen species (ROS) and cytochrome C that renders vulnerability to apoptosis (24). ROS production is highly correlated with PV concentration where alterations in redox homeostasis leads to a cascade of events that contribute to pathophysiological alterations in neurodevelopmental disorders (25, 26).

Parvalbumin is downregulated in various neurological conditions, such as in ASD Spectrum Disorder (ASD), a neurodevelopmental disorder characterized by deficits in social interaction and stereotypical behavior. Alterations of PV in the human ASD brain suggest that PV changes may promote an ASD like phenotype. PV-/- mice that constitutively lack PV expression showed ASD-like behavioral deficits, also suggesting that absent PV expression is sufficient to cause behavioral abnormalities (27). Genes, such as En2, *Mecp2, Fmr1, Cntnap2, Shank1 and Shank 3, Ube3, Nlgn3*, and K_V_3.1b, are implicated in ASD development and based on evidence derived from KO models are also believed to contribute to PV+ interneuron dysfunction (28–37). The knockout animal of the *Fmr1* gene–responsible for Fragile X Syndrome, the knockout of the *Shank3* gene–responsible for the Phelan-Mcdermind Syndrome, and the knockout of the *Ube3a*–responsible for Angelman syndrome, experience neuronal hyperexcitability, presumably because of impaired PV+ interneuron function (38–40). The *Nlgn3* animal model of ASD presents with decreased excitability of fast spiking interneurons and dysfunction of gamma oscillations, which are necessary for memory and attention (41). And the PV+ interneuron voltage-gated potassium channel K_V_3.1b, responsible for regulating the fast-spiking properties of PV+ interneurons, is reduced in the cortex and thalamus of Valproic Acid (VPA)-exposed mice, a widely used model of ASD (8). These findings indicate that a series of genes that play a direct or indirect role in regulating PV levels and function, are implicated in ASD. In addition, many EEG studies in human show that ASD individuals have abnormal gamma oscillations (41–43). Due to the functional role of PV+ cells in generation of gamma oscillations, and the evidence found in human and animal studies, it has been speculated that gamma oscillations could potentially be a physiological biomarker for abnormal functioning of PV+ neurons.

## Parvalbumin+ chandelier cells

**Figure 1:**
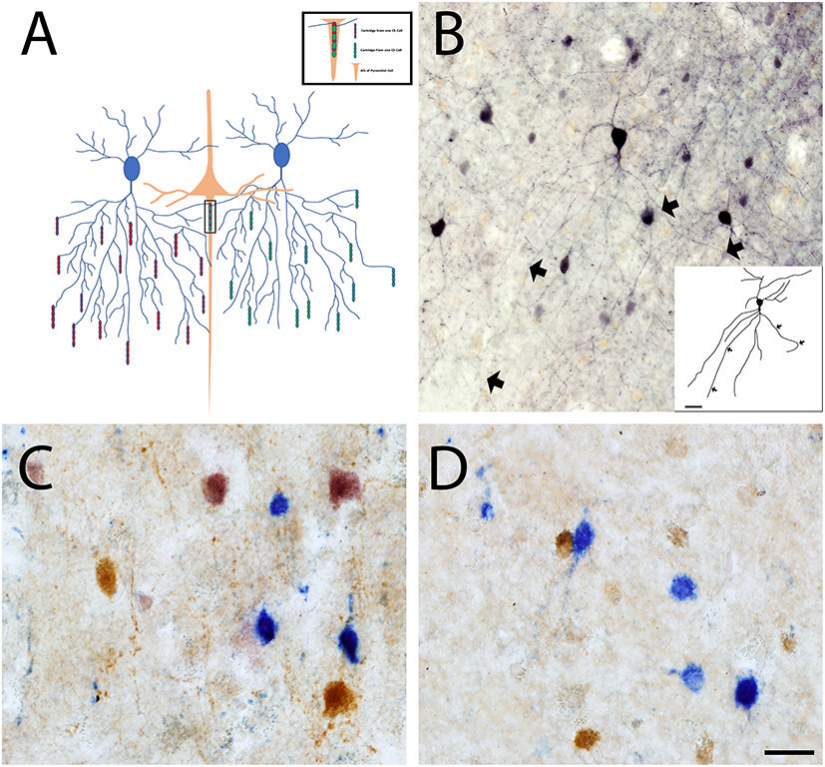
Chandelier cell connectivity and immunostaining. **(A)** Chandelier cells (blue) have a unique and structurally complex morphology characterized by vertically oriented axon terminal cartridges (red and green), that are perpendicular to the pial surface. Ch cells synapse onto the AIS of pyramidal neurons (orange) and can innervate up to 250 pyramidal cells at once, allowing for fine inhibitory control. **(B)** Ch cell in primary somatosensory cortex stained with an anti-PV antibody that labels the soma and proximal processes (BA3). Triple enzymatic stain of CR (Blue), CB (Brown) and PV (Pink) labeled interneurons reveals loss of PV+ interneurons in BA3 of both control **(C)** and ASD cortex **(D)**. Scale Bars = 20 μm.

Interneurons in the cortex exhibit a wide variety of morphological, physiological, and molecular characteristics. Interneurons can be classified based on the expression of specific molecular markers, among them fast-spiking interneurons can be recognized by their expression of PV. PV+ interneurons are GABAergic and modulate the output of pyramidal neurons by directly innervating their soma or the axon initial segment (AIS). In the cerebral cortex, PV+ interneurons include two distinct types, chandelier (Ch) cells and basket (Bsk) cells (44, 45). In mice, PV+ cells account collectively for approximately 40–50% of all interneurons in the cerebral cortex (46), with Bsk cells being more numerous and Ch cells making up less than 5% of all interneurons in the cortex. In other species, like Rhesus macaque, approximately 75% of all GABAergic neurons in the primary visual cortex are PV+ neurons (47). Ch cells and Bsk cells exhibit substantially different innervation properties and distinct firing properties. Physiologically, Bsk cells have a greater firing latency while Ch cells have a higher firing frequency and adaptation (48, 49). Anatomically, Bsk cells innervate the soma and proximal dendrites of pyramidal neurons, while Ch cells innervate the AIS of pyramidal neurons (44, 45). Basket cells are multipolar PV+ interneurons located throughout layers II–VI, while their prominent basket structures are mostly restricted to layer IV. Bsk cells establish multiple connections with the soma and proximal dendrites of pyramidal neurons in a manner that outlines the pyramidal cell body acquiring a basket-like shape. Bsk cells can also innervate other Bsk cells and non-PV+ GABA cells, like purkinje cells in the cerebellum (49–51). Basket cells are subdivided into small, large, and nest basket cells, that present with differential size, dendritic and axonal projections, firing properties, and the expression of additional molecular markers (52, 53). Ch cells are involved in the generation of gamma oscillations generated by the cortex (45, 54, 55). Specifically, synaptic inhibition from Ch cells controls the firing rate of pyramidal cells, synchronizes spikes within populations of neurons, and participates in cortical executive functions (52, 56, 57). While GABAergic input on dendrites originates from many types of interneurons, the AIS only receives input from Ch cells (58). Therefore, Ch cells play a central role in regulating the final output of excitatory pyramidal neurons. The terminal portions of Ch cell axons form vertical rows of synaptic boutons that resemble candlesticks and are known as cartridges (54, 59) (Figure 1). Ch cartridges express PV but are more easily detected by their expression of GABA transporter 1 (GAT1) (44, 60, 61). Each Ch cell has many cartridges lined with boutons and each cartridge can selectively innervate the AIS of a pyramidal neuron. A single pyramidal cell receives input from 1 to 4 Ch cells, while a single Ch cell innervates up to 250 pyramidal cells (62). Each Ch cell can therefore regulate the output of many pyramidal cells, and the loss of a single Ch cell signifies the loss of inhibitory AIS-mediated regulation of a great number of pyramidal cells belonging to several cortical mini-columns. The loss of a few Ch cells would critically impair pyramidal cell output and cerebral cortex function (58).

Ch cells are present in most cortical areas and play an important inhibitory and regulatory role in each of them. However, they are not homogeneously distributed but they differ in density by cortical area and cortical layer (63, 64). PV+ Ch cells are less prominent in deeper layers and some Ch cells co-express calbindin (CB) in human (65). These differences suggests that there is a region specific anatomical and functional organization. In human and other primates there is a higher density of cartridges innervating the AIS in association areas than in primary sensory areas (63, 66, 67). Only one study has systematically looked at the relative abundance of Ch cells in different areas of the human cortex and did so by mapping GAT1+ Ch cartridges (44). The lowest density of GAT1+ cartridges was in primary and secondary visual (BA17 and BA18) and somatosensory areas (BA3b and BA1). In contrast, there was a moderate density in primary motor cortex (BA4) and associative frontolateral areas (BA45 and 46), whereas other associative frontolateral cortex (BA9 and BA10), frontal orbital cortex (BA11, BA12, BA13, BA14, and BA47), associative temporal cortex (BA20, BA21, BA22, and BA38), and cingulate cortex (BA24 and BA32) displayed the highest density of GAT1+ cartridges (44). Despite these differences, the laminar distribution of GAT1+ cartridges was similar in most cortical areas. The highest density was in layer II, followed by layers III, V, VI, and IV. In most cortical areas, the density of GAT1+ cartridges was correlated with the neuronal density (44). PV+ Ch cell interneurons in the cerebral cortex originate subcortically in two waves of proliferation in the medial ganglionic eminence (MGE) during prenatal development. A small pool of radial glial cells that at embryonic day (E)10 are restricted to the caudal MGE generate a small set of layer V-VI and II-III Ch cells. A second and bigger wave at E12 gives rise to Ch cells across layers II-VI and occupies the entire rostral caudal region of the MGE (68). MGE-derived cells then migrate tangentially to the cortex in a spatiotemporal and time sensitive manner, reaching the cortex at E18-P0 (69–71). Once in their final cortical destination, they mature into PV+ Ch cells. Perturbations during the periods of proliferation, migration, and maturation, can result in cell death or malfunction.

While Bsk cells are present in many subcortical regions of the brain, Ch cells outside the cortex have only been identified in the hippocampal formation and the amygdaloid complex. The hippocampal formation is a brain structure involved in the generation, organization, and storage of new memories. Ch cells in the hippocampal formation are similar in morphology, marker expression, and electrophysiological properties to those in the neocortex. In the dentate gyrus (DG), PV+ cells are mostly located within or adjacent to granular zone (GZ) and are larger in size than the surrounding PV- granule cells (72). Most of the PV+ neurons in CA1 and CA3 are in the strata pyramidale and oriens, while a small number are present in stratum lucidum and in stratum radiatum, and rarely in stratum moleculare. In the DG, GAT1+ cartridges are located in the GZ and in the polymorphic layer (72). In the CA, GAT1+ cartridges are located in stratum pyramidale of CA1 and CA3 and are sparse in the stratum oriens. In CA2 and CA4, cartridges are only occasionally present (73–75). Ch cells in CA synapse and inhibit the AIS of pyramidal cells regulating their final output. Ch cells in the DG synapse on to the AIS of the granular cells in the GZ, also regulating their final output (74). The amygdaloid complex (amygdala) is involved in processing emotional responses and affective states (76, 77). Ch cells in the amygdala can be identify based on the morphology of their axonal terminals. Ch cells in the amygdala are similar in morphology, marker expression, and electrophysiological properties to those in the neocortex and hippocampal formation. PV+ interneurons are restricted to the basolateral (BLA) nuclei, with highest density of the cartridges in the lateral (75%), whereas the basal nucleus contains only 20% (76, 78). PV+ Ch cells synapse and inhibit the AIS of the pyramidal cells in the BLA nuclei controlling their final output. Alterations in the PV+ Ch cell population in the cerebral cortex, hippocampal complex, and amygdaloid complex may be a potential characteristic of the ASD brain.

## Parvalbumin+ chandelier cells in ASD

To investigate whether there are changes in the number or proportion of interneuron subpopulations in the prefrontal cortex in ASD, our group collected data using a straightforward approach to classify interneurons based on their expression of the calcium-sequestering proteins PV, CB, and calretinin (CR). Previous studies have used this method to comprehensively identify interneuron subpopulations in the cortex of mammalian species, and to identify interneuron subpopulations in human cortical tissue obtained from patients with certain conditions, like ASD (79, 80). We quantified PV+ cells in 10 ASD and 10 CT sex-and age-matched subjects and found that there is a decrease in the ratio of PV+ cells vs. the total number of interneurons, but no change in the ratio of CB+ cells or CR+ cells vs. the total number of interneurons in ASD compared to control cases (Figure 1) (81). Using an exclusion method, in which almost all Ch cells express PV but not the perineural net protein carbohydrate N-acetylgalactosamine, while human Bsk cells express both PV and N-acetylgalactosamine, we found a decrease in the number of PV+ in ASD that is attributable to a decrease in the number of Ch cells (82). Using a different cohort of subjects, we also found a decreased number of GAT1+ cartridges in the prefrontal cortex in ASD that was similar to the decreased number of PV+ cells in ASD, corroborating our previous data (60). In addition, we reported a decreased amount of GABA_A_Rα_2_ in the pyramidal cell AIS (83) - target of Ch cells synapses – and a reduced Ch bouton size, likely indicating a decreased synapsis strength – in the prefrontal cortex in ASD and concluded that Ch cells play an important role in the cortical circuitry dysfunction in ASD. Accordingly, the Barba's group examined PV+ and CB+ neurons in postmortem BA9 tissue from subjects with ASD (*n* = 2 ASD, 30 and 44 years, and *n* = 2 controls), and found a decrease in the ratio of PV to CB inhibitory neurons (84). We are not aware of any other data on Ch cells in ASD. We concluded that PV+ Ch cell number is decreased in the prefrontal cortex in ASD.

## Parvalbumin and parvalbumin+ cell alterations in other neuropsychiatric disorders

As in the case of ASD, changes in the morphology and/or function of PV+ interneurons have also been reported in schizophrenia, epilepsy, bipolar disorder, and depression (58, 85–89). During development subjects with schizophrenia exhibit elevated mRNA levels for the transcription factors *MafB* and *Lhx6*, expressed in PV+ cell progenitor cells that migrate from the MGE into the developing cortex (90–92). Activation of the Il-6/NOX2 pathway in a mouse model of schizophrenia elevates oxidate stress, perturbs the normal development and maturation of PV+ cortical interneurons, and contributes to the emergence of schizophrenia like behavioral deficits (93). PV+ interneurons in schizophrenic patients present with lower mRNA levels of PV and GAD67 (94–97). GAD67 protein levels have also been shown to be reduced in schizophrenia (98). However, GAD67 protein levels were not altered in the axon cartridges of PV chandelier cells. GAD67 mRNA levels were only reduced in Bsk cells suggesting that GAD67 changes may be cell specific and differentially affected in schizophrenia (98). In addition to mRNA levels, the levels of PV protein are lower in PV+ Bsk boutons and the density of PV+ Ch cell cartridges is decreased in the prefrontal cortex in schizophrenia (89). While a few studies have previously reported deficits in PV neuron density in the dorsolateral prefrontal cortex, it may be possible that lower PV immunoreactivity levels may have rendered these PV+ neurons below the detection threshold (99–101). In addition, Ch cell GABA transmission is altered in schizophrenia at both the pre and post synaptic level. At the presynaptic level there is a reduction of GAT1 immunoreactivity in the axon terminals of Ch cells within the PFC (85, 89). Despite this GAT1 reduction, the levels of vGAT bouton protein were unaltered in schizophrenia (102). These results, combined with the GAD67 alterations highlighted above underlies the importance of assessing multiple markers in a cell specific manner to better understand the extent of GABAergic signaling changes at the presynaptic level.

At the post synaptic level, there is an increase of GABA_A_Rα_2_ at the AIS of pyramidal neurons (88). Moreover, the density of ankyrin G, an adaptor molecule responsible for the recruitment and stabilization of sodium channels at the AIS, is also decreased by 19% in brains with schizophrenia (103, 104). Knockdown of *L1CAM* in mice, a neural adhesion molecule that is linked to schizophrenia, results in a decrease of pyramidal AIS innervation by Ch cells (105, 106). In line with these findings, transcriptional levels of *Erbb4*, a known susceptibility gene for schizophrenia that is primarily expressed in PV+ neurons and highly active during early neurodevelopment, is decreased in Ch cells in the prefrontal cortex (107–113). Mice that lack *Erbb4* in fast-spiking interneurons, have synaptic defects, while Erbb4 -/- mice are characterized by impaired behavior and GABA release (114, 115). Genetic recovery of the gene in ErbB4 -/- mice at the adult stage ameliorates these deficits demonstrating the role of ErbB4 mediated signaling in establishing and maintaining optimal Ch cell function (107). Activation of the Il-6/NOX2 pathway in a mouse model of schizophrenia elevates oxidate stress, perturbs the normal development and maturation of PV+ cortical interneurons, and contributes to the emergence of schizophrenia like behavioral deficits (93). Together these findings demonstrate that the synaptic integrity of Ch cell pyramidal connection is compromised in schizophrenia.

Half of the cases of ASD also present with epilepsy and the cortex in brains with epilepsy also present significant reductions in mRNA levels of PV. Post-mortem studies in brains of patients with temporal lobe and cryptogenic frontal lobe epilepsy present patches of decreased PV+ immunoreactivity in the neocortex (116), and the human epileptic peritumoral neocortex has a loss of inhibitory synapses on the soma and AIS of pyramidal neurons (117). A more recent human study showed that there was an increased density of PV+ Bsk cell boutons in the dentate gyrus, while the density of PV+ Ch cell boutons increased significantly in subjects with hippocampal sclerosis, a common pathology encountered in mesial temporal love epilepsy (118). Interestingly, bouton densities were not different between epileptic subjects without hippocampal sclerosis and matched controls. In monkeys with cortical focal epilepsy there is a reduction of Ch cell axons at the epileptic foci (119). Moreover, presentation of anxiety like behaviors, coupled with decrease in density of PV+ interneurons, GAD65 containing synaptic terminals and increases in GABA_A_R β3 subunit are all phenotypic traits observed across multiple pilocarpine-models of temporal lobe epilepsy (120, 121). Accordingly, pharmaco-genetic activation of hippocampal PV interneurons alleviates the severity of seizure onset in chronic epilepsy models (97). These findings suggest that synaptic connectivity is affected in individuals with epilepsy and may contribute to dysregulated inhibitory signaling. Individuals with bipolar disorder also present reduced PV messenger levels in the prefrontal cortex and hippocampus (122, 123), and reduced PV+ interneuron density in the prefrontal and entorhinal cortex, and parasubiculum (124, 125). There is also a decrease in GABA receptor levels in the PFC. The density of neurons containing messenger for GAD65 and GAD67 in the hippocampus is decreased by 45%, and the expression of GAD67 protein is reduced 50% in the prefrontal cortex and cerebellum in bipolar disorder brains (126, 127). Brains from people with depression also present a reduction in PV+ immunostaining in layer VI of the dorsolateral prefrontal cortex (128, 129), and animal models of depression present with chronic stress-induced depressive-like behavior and reduced density of hippocampal PV+ interneurons (99). In addition, ketamine's antidepressant properties are mediated in part through downregulation of NRG1-ErbB4 singling in PV+ interneurons in the rat brain (130). It is well established that stress is a risk factor that exacerbates the development of these disorders, likely in part through the perturbation of normal PV+ interneurons development and function (98–100). Animal models exposed to early stress during development have increased anxiety like behavior coupled with a decrease in PV+ interneuron density (98, 101, 118). Administration of a neurokinin-1 receptor (NK_1_R) antagonist prior to chronic stress exposure completely prevent the stress-induced reduction of the number of PV interneurons in mice (131). And rearing behavior in an enriched environment augment the number of PV+ interneurons in the basolateral amygdala and decrease anxiety like behavior in young male rats (132). These findings demonstrate that PV function is also impaired in neuropsychiatric disorders like temporal lobe epilepsy, depression and bipolar disorder, at both pre and post synaptic levels.

Overall, there is evidence that parvalbumin and PV+ interneurons in the cortex have a role in ASD, but also in other psychiatric, neurodevelopmental, and mood disorders, including epilepsy, schizophrenia, bipolar disorder, and depression.

## Author contributions

VM wrote the manuscript. PJ wrote the manuscript and obtained images for figure. All authors contributed to the article and approved the submitted version.

## Funding

This study was funded by the National Institute of Mental Health MH094681 and Shriners Hospitals for Children.

## Conflict of interest

The authors declare that the research was conducted in the absence of any commercial or financial relationships that could be construed as a potential conflict of interest.

## Publisher's note

All claims expressed in this article are solely those of the authors and do not necessarily represent those of their affiliated organizations, or those of the publisher, the editors and the reviewers. Any product that may be evaluated in this article, or claim that may be made by its manufacturer, is not guaranteed or endorsed by the publisher.
